# Whole-Genome Optical Mapping and Finished Genome Sequence of *Sphingobacterium deserti* sp. nov., a New Species Isolated from the Western Desert of China

**DOI:** 10.1371/journal.pone.0122254

**Published:** 2015-04-01

**Authors:** Chao Teng, Zhengfu Zhou, István Molnár, Xinna Li, Ran Tang, Ming Chen, Lin Wang, Shiyou Su, Wei Zhang, Min Lin

**Affiliations:** 1 Biotechnology Research Institute, Chinese Academy of Agricultural Sciences, Beijing, P.R. China; 2 Natural Products Center, School of Natural Resources and the Environment, University of Arizona, Tucson, Arizona, United States of America; 3 Key Laboratory of Resource Biology and Biotechnology in Western China, Ministry of Education, Northwest University, Xi’an, P.R. China; The University of Hong Kong, HONG KONG

## Abstract

A novel Gram-negative bacterium, designated ZW^T^, was isolated from a soil sample of the Western Desert of China, and its phenotypic properties and phylogenetic position were investigated using a polyphasic approach. Growth occurred on TGY medium at 5–42°C with an optimum of 30°C, and at pH 7.0–11.0 with an optimum of pH 9.0. The predominant cellular fatty acids were summed feature 3 (C_16:1_ *ω7c*/C_16:1_ *ω6c* or C_16:1_ *ω6c*/C_16:1_ *ω7c*) (39.22%), iso-C15:0 (27.91%), iso-C_17:0_ 3OH (15.21%), C_16:0_ (4.98%), iso-C_15:0_ 3OH (3.03%), C16:0 3OH (5.39%) and C_14:0_ (1.74%). The major polar lipid of strain ZW^T^ is phosphatidylethanolamine. The only menaquinone observed was MK-7. The GC content of the DNA of strain ZWT is 44.9 mol%. rDNA phylogeny, genome relatedness and chemotaxonomic characteristics all indicate that strain ZW^T^ represents a novel species of the genus *Sphingobacterium*. We propose the name *S*. *deserti* sp. nov., with ZW^T^ (= KCTC 32092T = ACCC 05744T) as the type strain. Whole genome optical mapping and next-generation sequencing was used to derive a finished genome sequence for strain ZW^T^, consisting of a circular chromosome of 4,615,818 bp in size. The genome of strain ZW^T^ features 3,391 protein-encoding and 48 tRNA-encoding genes. Comparison of the predicted proteome of ZW^T^ with those of other sphingobacteria identified 925 species-unique proteins that may contribute to the adaptation of ZW^T^ to its native, extremely arid and inhospitable environment. As the first finished genome sequence for any *Sphingobacterium*, our work will serve as a useful reference for subsequent sequencing and mapping efforts for additional strains and species within this genus.

## Introduction

The genus *Sphingobacterium* was originally proposed by Yabuuchi [1] for bacteria whose membranes contain high concentrations of sphingolipids. Sphingobacteria are Gram-negative rods that contain MK-7 as their predominant isoprenoid quinone, and the GC content of DNA is ranging from 35 to 44 mol% [2]. At present, the genus *Sphingobacterium* encompasses 32 validly published species names. However, this number continues to increase as novel *Sphingobacterium* strains are isolated from various samples of soil, compost [3] and sludge [4], and even from human clinical specimens [5].

Bacterial genome sequencing is rapidly emerging as the most important source of information for microbial taxonomy. For example, determination of the whole genome sequence of a newly isolated strain allows the calculation of average nucleotide identity (ANI) scores, providing global comparisons of the new strain with previously isolated strains whose genome sequences are deposited in databanks. These ANI scores will probably serve as the next-generation gold standard for species delineation [6].

Whole-genome optical mapping (WGM) is an important tool for bacterial genome sequencing. It includes the assembly of whole-genome restriction endonuclease maps by digesting immobilized DNA molecules and determining the sizes and the order of the produced fragments [7, 8]. WGM offers a relatively economical and rapid method of assessing rates of mutation and recombination, and may be used to determine the taxonomic position and the evolutionary history of a particular strain. The value of WGM as a strain-typing tool in *E*. *coli* outbreaks has been documented, and the method has recently been applied to subtyping analysis [9–11]. Furthermore, WGM allows classical genetic sequencing to better target particular genomic regions and alterations in genome structure [12]. Importantly, sequencing the genome of a bacterial strain with WGM also facilitates the future annotation of genome sequences of additional strains from the same genus. This is because WGM allows the rapid, direct transfer of any gene- or genome features from the initial, voucher strain to the newly sequenced strain. Whole-genome restriction maps created to designate novel species also allow the rapid resolution of sequence assembly problems, permit completion of the genome, and correct large inversions in genome assemblies. The accuracy of a genome assembly can also be further assessed by fluorescence in situ hybridization (FISH) analysis.

This communication describes the phenotypic and genomic properties of a novel *Sphingobacterium* strain, *S*. *deserti* ZW^T^ that was isolated from the Western Desert of China. We have created a *Bam*HI whole genome optical map for this strain, and used the WGM as a scaffold for sequence assembly and as a tool for the validation of the finished sequence. Since *S*. *deserti* ZW^T^ is the first strain within the *Sphingobacterium* genus with a fully assembled and finished genome sequence, our work will serve as a useful reference for any future genomic studies of strains from this genus. Further, the genomes of microorganisms isolated from desert ecosystems may hold clues to the adaptation of microbial life to inhospitable environmental conditions that lead to DNA damage due to high levels of solar irradiation, as well as protein denaturation due to desiccation and extreme and sudden temperature shifts. Thus, comparison of the genome sequence of *S*. *deserti* with those of other *Sphingobacterium* strains allowed the identification of species-unique genes and pathways that may be important to mediate the adaptations of this species to its extreme environment.

## Materials and Methods

### Ethics Statement

No specific permits were required for the locations or activities described in this work. The field studies did not involve endangered or protected species.

### Strain isolation

Strain ZW^T^ was isolated from a soil sample that was collected from the extreme arid environments of the Western Desert of China. The GPS coordinates for the sampling site were 42° 99.92’ N, 89° 12.63’ E. The soil sample was dispersed in TGY medium (1.0% tryptone (Oxoid Ltd., England), 0.5% yeast extract (Oxoid Ltd., England), 0.1% glucose (Xilong Chemical Co., Ltd., China)) and the culture was incubated for 2 days at 30°C with shaking at 200 rpm before dilution plating on TGY agar plates (30°C, 24 h) to isolate single colonies. The isolates were maintained at -80°C as suspensions in TGY medium supplemented with 20% glycerol (v/v, final concentration). Besides strain ZW^T^, a different *Sphigobacterium* strain was also isolated from the same soil sample and described as an isolate of *Sphingobacterium arenae* [13].

### Morphological, physiological and chemotaxonomical characteristics

A polyphasic approach was used to identify strain ZW^T^ and to determine its taxonomic position. Gram staining was conducted according to the method of Cowan [14] after growing ZW^T^ cells for 1 day on TGY agar. Colony morphology was examined for cultures grown on TGY agar for 48 h at 30°C. Cell morphology was determined using scanning electron microscopy (S-570; Hitachi; Japan). The growth of strain ZW^T^ at various temperatures (4, 10, 15, 20, 25, 30, 33, 37, 40, 42, 45 and 50°C) and pH (5.0–10.0, in increments of 0.5 pH units) was assessed in TGY after 5 days, with the pH value being adjusted using the appropriate buffers as described by Dimitry *et al*. [15]. Salt tolerance, catalase activity, oxidase activity and substrate utilization were determined using the method of Jiang [13]. The isoprenoid quinone test was performed as described by Zhou [16]. *S*. *composti* and *S*. *arenae* were used as reference strains for menaquinone identification. For the analysis of cellular fatty acids, both strain ZW^T^ and *S*. *composti* 4M24^T^ were harvested after 48 h of growth on TGY medium (pH 9.0) at 30°C. Identification of fatty acids were conducted according to Yoo *et al*. [3, 16]. Sphingolipid analysis was performed according to Yabuuchi [17]. Physiological characterization and additional biochemical tests were performed according to the method described by Zhu [18].

### GC content, PCR amplification, sequencing and phylogenetic analysis

The genomic DNA of strain ZW^T^ was prepared using the TIANamp bacterial DNA isolation kit (Tiangen). The GC content of the DNA was determined according to the procedure of Zhou [16]. DNA-DNA hybridization experiments were performed according to the method developed by Yuan [19]. PCR amplification and sequence analysis of the 16S rRNA gene has been previously described in detail [16]. Phylogenetic dendrograms, which displayed substantially identical topologies, were constructed using the neighbor-joining [20], the maximum likelihood and the maximum parsimony methods [21], with bootstrap values calculated from 1,000 re-samplings.

### Optical map construction

Optical maps were prepared using Argus (OpGen) according to previously described methods [22]. Protoplasts of ZW^T^ were prepared by enzymatic digestion of the cell wall [11]. High molecular weight DNA was prepared by embedding protoplasts in low melting temperature agarose plugs, followed by treatment with lysing solution. The genomic DNA was recovered after thoroughly rinsing the plugs in TE, followed by melting the plugs at 42°C and subsequent treatment with β-agarase. Individual molecules of the high molecular weight DNA were then immobilized onto Optical Chips, digested with *Bam*HI (New England Biolabs), and stained with a fluorescent kit (OpGen). High resolution single-molecule restriction maps were created by measuring the sizes of the restriction fragments after image capture on an automated fluorescent microscope system (Argus; OpGen; USA). Collections of single molecule maps were then assembled to produce a whole-genome ordered restriction map.

### De novo assembly of the optical map

The mapset (total dataset generated from a single run) was filtered for minimum molecule size (> 150 kb), minimum fragments per molecule (>12) and minimum molecule quality (>0.5). The data from each MapCard were combined for the final assembly. The assembly was conducted after removal of default circularization parameters. Partial assembly results were saved when three contigs became apparent with >10 molecules each. Contigs were separated and individually reassembled against the original mapset using the “Find Hits” feature; they were considered “finished” when no additional molecules could be added by subsequent reassemblies. Overlapping single-molecule maps were assembled using the Optical Map Assembler software (OpGen) to create a circular map spanning the entire genome with approximately 30-fold coverage.

### Sequence-to-map comparison

Comparisons between optical maps and sequence contigs were performed using the MapSolver software (OpGen Technologies, Inc.). Sequence FASTA files were converted to *in silico* restriction maps for direct comparison to the optical maps. Alignments were generated with a dynamic programming algorithm that finds the optimal location or placement of a sequence contig by first performing a global alignment of the sequence contig against the optical map. Local alignment analyses were also performed to compare segments of the sequence contigs to the optical map. Finally, the MapSolver software was used to place the predicted restriction maps of large contigs (>50 kb) resulting from the next generation sequencing (NGS) assembly on the optical map scaffold, enabling gaps between contigs to be predicted and filled.

### Genome sequencing and analysis of strain ZW^T^

The genome sequence of strain ZW^T^ was determined using Illumina whole genome shotgun sequencing technology. A total of 5,477,992 reads were obtained with a total read length of 504 Mb, representing a 109-fold coverage of the genome. Short reads generated from the Illumina pair-end library were assembled with the Velvet assembler to yield a draft genome assembly consisting of 21 scaffolds. The finished genomic assembly was generated with whole-genome optical mapping as described above. Genome sequencing and restriction map construction were performed at Tianjin Biochip Corporation.

Putative protein-coding sequences were predicted using Glimmer. Functional annotations were based on BLASTP analyses against the KEGG, Pfam, COGs and NCBI non-redundant (nr) protein sequence databases [23]. tRNA genes were directly predicted with tRNAscan-SE [24], and the rRNA genes were identified with RNAmmer [25].

### Orthology and phylogenetic analysis

Predicted protein sequences were searched against the nr database of NCBI by an all-versus-all BLASTp with a threshold value of E ⩽1e-5, and then clustered by orthoMCL [26] with an inflation value of 1.5. Multiple sequence alignments of the orthologous proteins were produced using MUSCLE (v3.6) [27, 28], and concatenated into a single multiple sequence alignment with an in-house Perl script. Neighbor-joining phylogeny was then reconstructed using MEGA (v5).

### Average nucleotide identity

Average nucleotide identity (ANI) was calculated by the JSpecies program version 1.2.1 using ANIb with default settings [29].

## Results and Discussion

### *S*. *deserti* sp. nov.

**Fig 1:**
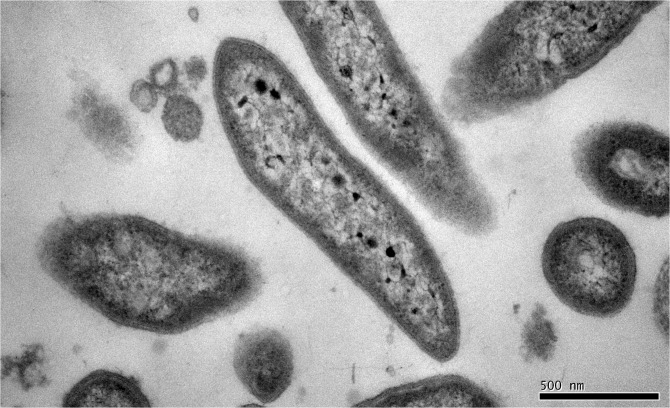
Electron micrograph of the cell morphology of strain ZW^T^.

The cells of strain ZW^T^ are Gram-negative, non-motile, non-spore-forming, strictly aerobic rods, approximately 0.6–1.7 μm in length and 0.3–0.6 μm in diameter (Fig 1). After 48 h of incubation on TGY, ZW^T^ colonies were 3–5 mm in diameter, lemon yellow, convex, circular, smooth and entire. The optimum growth temperature is 30°C (range: 5–42°C), and the optimum pH is 9 (range: pH 7.0–11.0). Growth occurs at NaCl concentrations ranging from 0 to 1%. Cells of strain ZW^T^ are positive for catalase and oxidase activities, but negative for urease. Casein, starch, gelatin, Tween 20 and Tween 80 are not hydrolyzed. Cells of strain ZW^T^ do not show L-arginine dihydrolase activity, and are negative for citrate utilization, nitrate reduction, and the Voges-Proskauer reaction. They are able to assimilate D-glucose, D-mannose, D-maltose, and L-arabinose, but not xylose. Acid is produced from D-mannitol and L-arabinose, but not from D-fructose, D-xylose or salicin (Table 1). The cells of strain ZW^T^ exhibit resistance to kanamycin (50 μg/mL), ampicillin (50 μg/mL), hygromycin (50 μg/mL) and spectinomycin (35μg/mL), but are sensitive to rifampicin (50 μg/mL), chloramphenicol (50 μg/mL) and tetracycline (50 μg/mL). The predominant cellular fatty acids are summed feature 3 (C_16:1_ *ω7c*/C_16:1_ *ω6c* or C_16:1_ *ω6c/*C_16:1_ *ω7c*) (39.22%), iso-C15:0 (27.91%), iso-C_17:0_ 3OH (15.21%), C_16:0_ (4.98%), iso-C_15:0_ 3OH (3.03%), C16:0 3OH (5.39%) and C_14:0_ (1.74%). This fatty acid profile is similar to those of several reference strains of sphingobacteria that were grown under identical conditions (Table 2), suggesting that this newly isolated bacterium belongs to the genus *Sphingobacterium*. The polar lipid profile of strain ZW^T^ includes phosphatidylethanolamine and several unidentified polar lipids (S1 Fig). Both strain ZW^T^ and *S*. *spiritivorum* contain sphingolipids (S2 Fig), which are a distinct feature of members of the genus *Sphingobacterium*. The menaquinone of strain ZW^T^ is MK-7 (>99%), consistent with all known members of the family *Sphingobacterium*. The GC content of the DNA of strain ZW^T^ is 44.9 mol%, slightly higher than that of other *Sphingobacterium* strains.

**Table 1:** Differential phenotypic characteristics of strain ZW^T^ and closely related *Sphingobacterium* species.

**Characteristic**	**1**	**2**	**3**	**4**	**5**	**6**	**7**	**8**	**9**
**Temperature range (°C)**	5–42	10–42	10–40	10–42	10–40	10–40	11–39	10–42	15–42
**Nitrate reduction**	-	-	-	-	-	-	-	-	-
**Urease**	+	-	+	+	ND	-	-	-	-
**Hydrolysis of**									
**Casein**	-	-	-	-	-	-	ND	-	-
**Gelatin**	-	-	-	-	ND	+	-	-	ND
**Tween 80**	-	+	+	-	+	-	+	ND	ND
**Acid production from**									
**D-Fructose**	-	+	+	-	-	-	+	ND	ND
**D-mannitol**	+	-	+	-	-	-	-	-	-
**L-Arabinose**	+	+	-	+	+	-	+	-	-
**Salicin**	-	+	+	-	+	+	ND	ND	ND
**DNA GC content (mol%)**	44.85	42.3	39.0	44.2	40.2	40.6	41.0	38.7	36.6

Strains: 1, *Sphingobacterium deserti* sp. nov. ZW^T^; 2, *S*. *composti* 4M24^T^ (data from this study); 3, *S*. *spiritivorum* JCM 1277^T^ (data from this study); 4, *S*. *arenae* H-12^T^ (data from this study); 5, *S*. *shayense* HS39^T^ [30]; 6, *S*. *nematocida* M-SX103^T^ [31]; 7, *S*. *bambusae* IBFC2009^T^ [32]; 8, *S*. *daejeonense* TR6-04^T^ [33]; 9, *S*. *kyonggiense* KEMC 2241-005^T^ [34]. +, Positive;-, negative; ND, no data available.

**Table 2:** Fatty acid composition of strain ZW^T^ and related members of the genus *Sphingobacterium*.

**Fatty acid**	**1**	**2**	**3**	**4**	**5**	**6**	**7**	**8**	**9**
**C**_**14:0**_	1.7	-	1.0	1.4	1.3	0.7	2.0	-	1.2
**C**_**16:0**_	5.0	2.2	3.5	7.3	3.5	4.5	1.8	3.4	3.2
**C**_**16:0**_ **3-OH**	5.4	1.2	2.7	4.8	2.4	3.3	15.2	-	6.7
**Iso-C**_**15:0**_	27.9	29.5	30.1	27.2	28.6	33.3	22.4	45.6	27.8
**Iso-C**_**15:0**_ **3-OH**	3.0	2.3	2.2	3.1	2.5	2.2	5.8	1.5	1.2
**Iso-C**_**17:0**_ **3-OH**	15.2	19.7	12.5	15.4	13.5	13.4	12.8	16.6	12.5
**Summed feature 3**	39.2	37.5	42.7	37.8	37.0	35.0	34.4	-	29.6

Strains: 1, *Sphingobacterium deserti* sp. nov. ZW^T^; 2, *S*. *composti* 4M24^T^ (data from this study); 3, *S*. *spiritivorum* JCM 1277^T^ (data from this study); 4, *S*. *arenae* H-12^T^ (data from this study); 5, *S*. *shayense* HS39^T^ [30]; 6, *S*. *nematocida* M-SX103^T^ [31]; 7, *S*. *bambusae* IBFC2009^T^ [32]; 8, *S*. *daejeonense* TR6-04^T^ [33]; 9, *S*. *kyonggiense* KEMC 2241-005^T^ [34]. Numbers represent percentages of the total fatty acids.–, not detected (<1%). Summed feature 3: C_16:1_ω7c / C_16:1_ω6c, or C_16:1_ω6c / C_16:1_ω7c.

**Fig 2:**
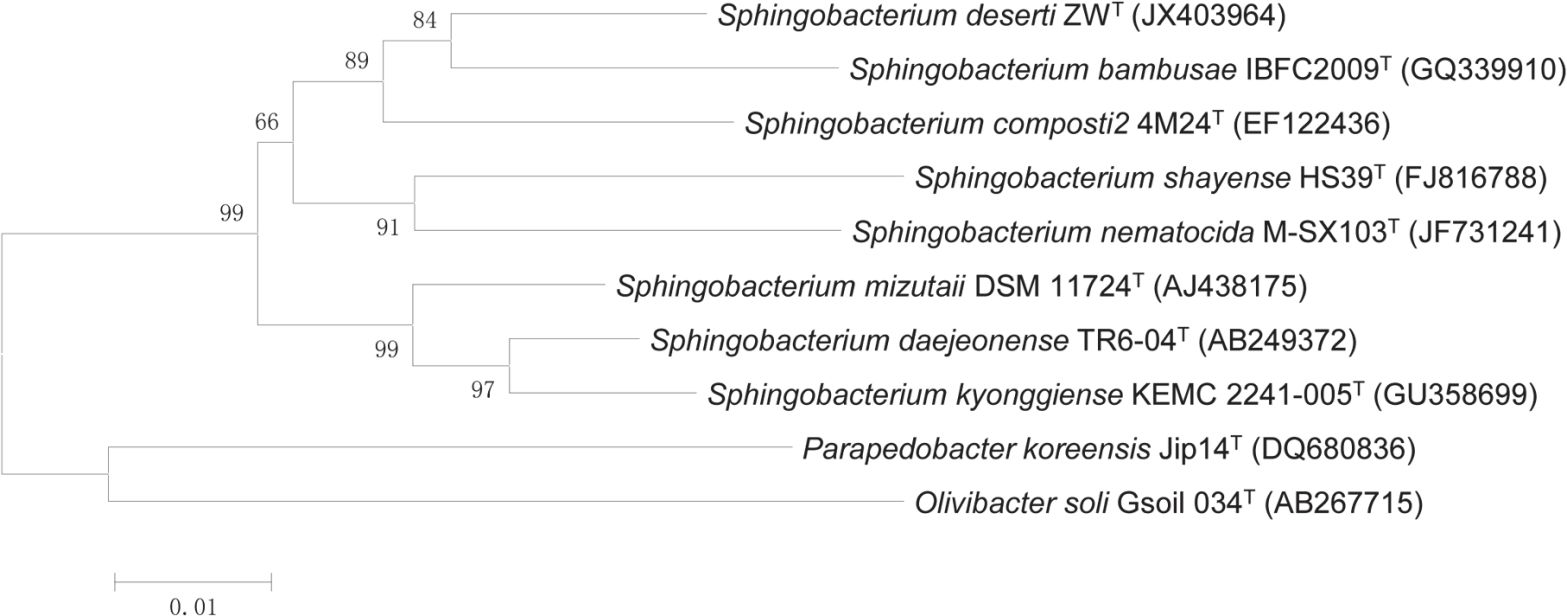
Neighbor-joining phylogenetic tree indicating the relationships between the strain ZW^T^ and other *Sphingobacterium* spp. Bootstrap values (expressed as percentages of 1,000 replications) of > 70% are shown at branch points. Bar, 0.01 substitutions per nucleotide position.

The 16S rRNA gene sequence of strain ZW^T^ (= KCTC 32092T = ACCC 05744T) is 1,311 bp in length (GenBank accession number JX403964). BLAST searches in the GenBank database and the EzTaxon server (http://www.ezbiocloud.net/eztaxon; [35]) indicated that strain ZW^T^ belongs to the genus *Sphingobacterium* of the phylum Bacteroidetes. The 16S rRNA gene of strain ZW^T^ exhibits the highest similarity to sequences from *S*. *bambusae* IBFC2009^T^ (95.65%), *S*. *composti* 4M24^T^ (95.57%), *S*. *lactis* WCC 4512^T^ (95.49%), *S*. *mizutaii* DSM 11724^T^ (95.11%), *S*. *daejeonense* TR6-04^T^ (95.11%), *S*. *kyonggiense* KEMC 2241-005^T^ (94.73%) and *S*. *nematocida* M-SX103^T^ (93.59%). DNA–DNA relatedness between strain ZW^T^ and *S*. *composti* 4M24^T^ is 36.3%. Phylogenetic analysis confirmed that strain ZW^T^ forms a coherent cluster with members of the genus *Sphingobacterium*, and an intra-genus clade with *S*. *bambusae* IBFC2009^T^ (Fig 2). According to Stackebrandt [36] and Wayne [37], 16S rRNA gene similarities that are lower than 97% and DNA–DNA relatedness values below 70% support the identification of bacterial isolates as belonging to a new species. The average nucleotide identity (ANI) of the genome sequence (see below) of strain ZW^T^ against the four other *Sphingobacterium* species for which genome sequences are publicly available ranged from 84.40% (with strain *S*. *thalpophilum* DSM 11723) to 84.94% (with strain *S*. *paucimobilis* HER1398). These ANI values are also considerably lower than the 95 to 96% threshold used to identify isolates as belonging to the same bacterial species [38, 39] (S1 Table). Thus, rDNA phylogeny (S3 Fig, S4 Fig), genome relatedness and chemotaxonomic characteristics all indicate that strain ZW^T^ represents a novel species within the genus *Sphingobacterium* (S5 Fig). We propose the name *S*. *deserti* sp. nov. (de.ser’ti. L. gen. n. *deserti* of a desert), with ZW^T^ (= KCTC 32092T = ACCC 05744T) as the type strain.

### Genome organization of strain ZW^T^

To further characterize this novel species, we sequenced the genome of strain ZW^T^ using WGM technology. The resulting whole genome optical map allowed the assembly of the first finished genome sequence, to the best of our knowledge, for any species within the genus *Sphingobacterium*.

**Fig 3:**
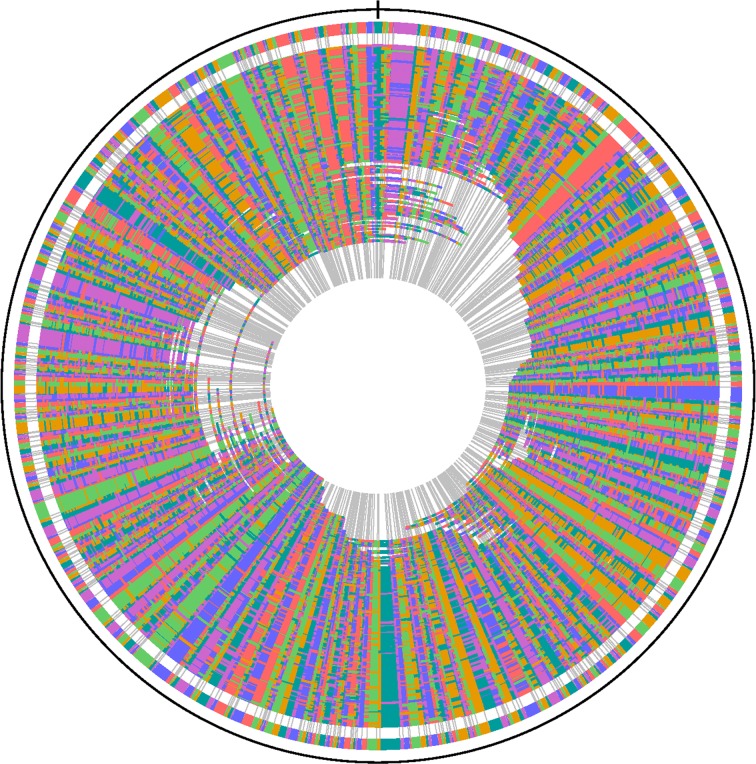
Optical mapping of the *Sphingobacterium deserti* sp. nov. ZW^T^ genomic DNA with *Bam*HI. The large chromosome restriction map was generated by shotgun optical mapping. The outer circle depicts the consensus map; the inner circles indicate the contigs from which the consensus map was generated. Colors are arbitrarily assigned to homologous overlapping fragments.

**Fig 4:**
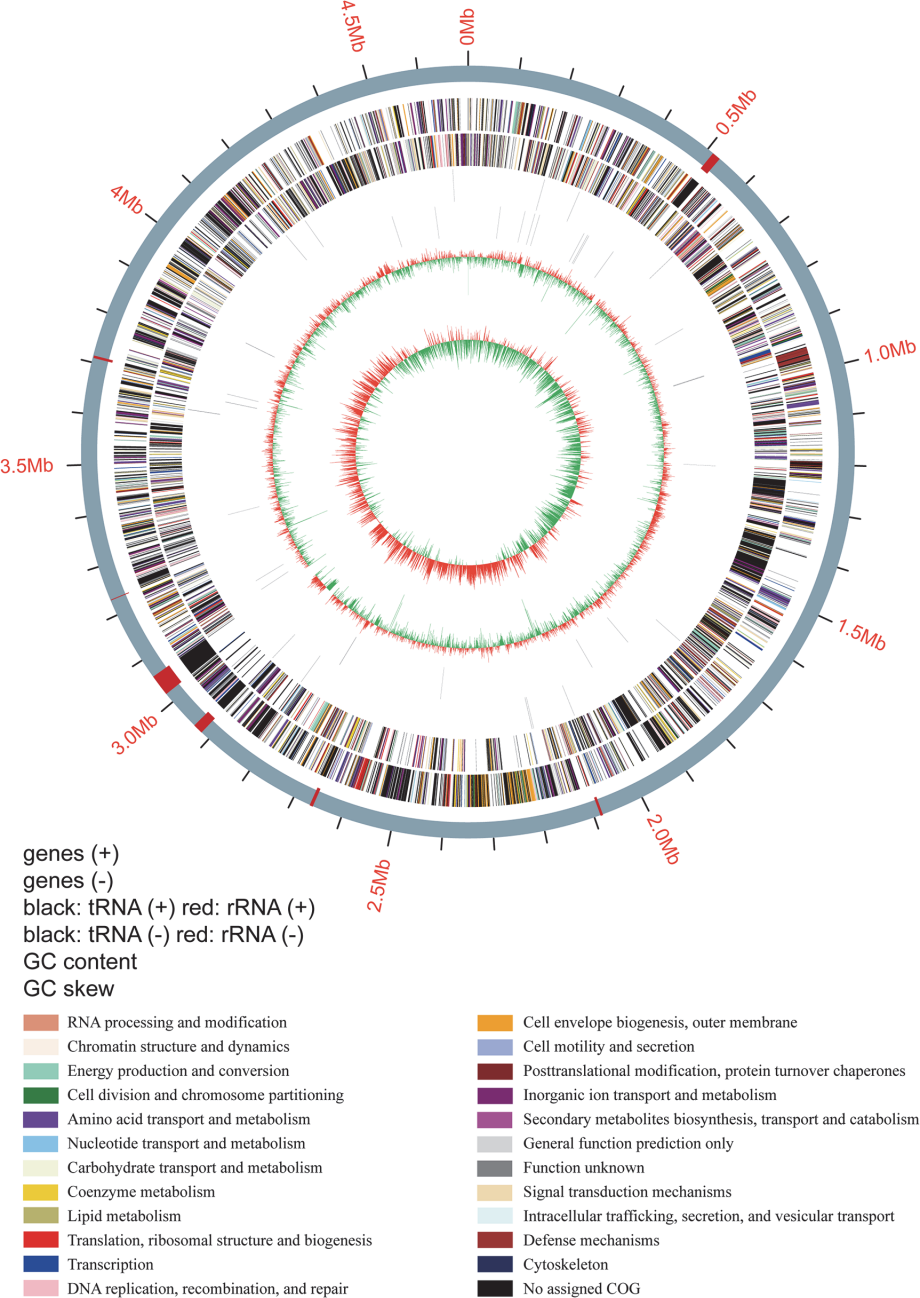
Genome map of *Sphingobacterium deserti* sp. nov. ZW^T^. Concentric tracks from the inside to the outside represent the GC nucleotide bias; the GC content; tRNA and rRNA genes on the reverse strand; tRNA and rRNA genes on the forward-strand; reverse-strand coding sequences (CDSs); and forward-strand CDSs.

The length of the circular chromosome of strain ZW^T^ was found to be 4,615,818 bp (Fig 3, GenBank accession number JJMU00000000). The GC content of the genome is 42.6 mol% (Fig 4), in agreement with the value (44.9 mol%) established by DNA renaturation kinetics [16]. This is within the range of the published GC contents of the four other *Sphingobacterium* species for which draft genome sequences are publicly available.

The genome of strain ZW^T^ contains 48 tRNA genes and several rRNA gene clusters, including single copies of the 16S and the 23S rRNA genes and duplicate copies of the 5S rRNA gene. An estimated 88.6% of the genome contains coding sequences (CDSs), and these CDSs are predicted to encode 3,391 putative proteins. The predicted proteins belong to 1,238 conserved orthologous groups (COGs) from 23 COG categories. In addition, 1,408 predicted proteins were also annotated using the KEGG Orthology System (S2 Table).

**Fig 5:**
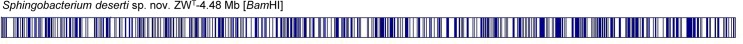
Whole-genome optical mapping barcode of *Sphingobacterium deserti* sp. nov. ZW^T^. Vertical lines represent restriction sites; distances between lines represent fragment sizes.

Although many *Sphingobacterium* strains have been isolated in the last 30 years, no finished genome assemblies have been generated for any of those strains. In addition, few chromosomal features have been reported for any *Sphingobacterium* genome. The genomic data and the WGM restriction barcode of strain ZW^T^ may facilitate the taxonomic characterization of further members of the genus *Sphingobacterium* (Fig 5). Strain ZW^T^ can also serve as a genome sequencing reference strain, with its WGM sequencing and annotation data available at NCBI for download and direct import into new draft genome sequences of other sphingobacteria.

### Evolution and comparative genomics

**Fig 6:**
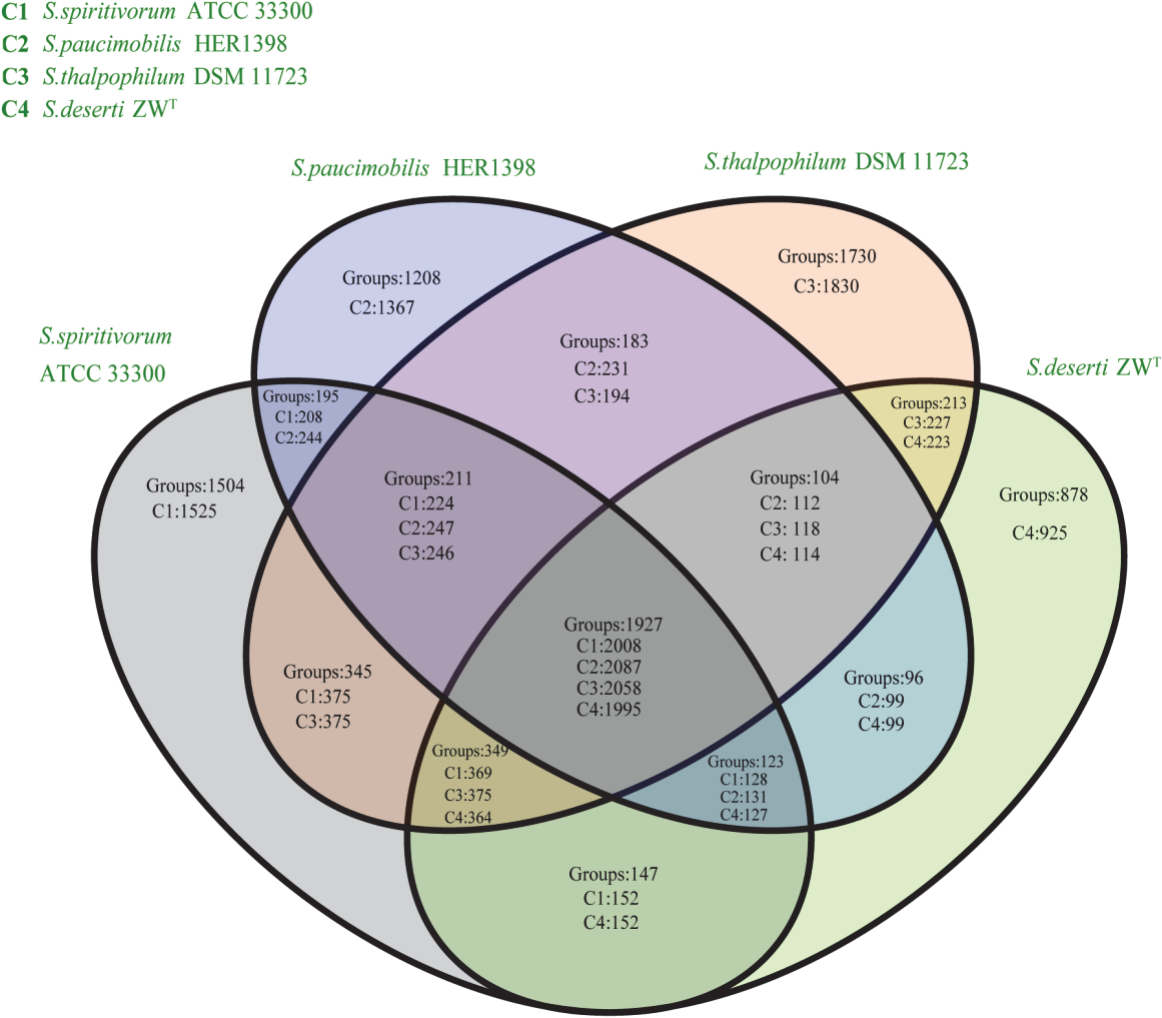
Venn diagram depicting orthologous groups of predicted proteins encoded in four sphingobacterial genomes. C1: *Sphingobacterium spiritivorum* ATCC 33300; C2: *Sphingobacterium paucimobilis* HER1398; C3: *Sphingobacterium thalpophilum* DSM11723; and C4: *Sphingobacterium deserti* ZW^T^.

We compared the predicted proteome of strain ZW^T^ with those of three other *Sphingobacterium* strains for which genome sequence data are available at NCBI (*S*. *paucimobilis*, *S*. *thalpophilum* and *S*. *spiritivorum*, Fig 6). These four sequenced *Sphingobacterium* strains share 1,927 orthologous protein groups. These common orthologous protein groups encompass the enzymes for the central carbon metabolism, and include the pentose phosphate pathway, the tricarboxylic acid cycle (TCA), the biosynthesis of amino acids, and the assembly of purine and pyrimidine nucleotides. Genes for this set of predicted pathways are well conserved in the genomes of the sequenced *Sphingobacterium* strains. Orthologous protein groups shared by ZW^T^ with only one of the other sphingobacterial strains were also identified, and contain 96 (*S*. *paucimobilis*), 213 (*S*. *thalpophilum*), and 147 (*S*. *spiritivorum*) groups, respectively. Importantly, we detected 925 species-unique predicted proteins belonging to 878 orthologous groups in strain ZW^T^. Many of these ZW^T^-specific genes are involved in transport systems, DNA repair and the biosynthesis of small molecules. These and other species-unique proteins may facilitate the adaptation of *S*. *deserti* to harsh arid lands environments [40].

### Genome-wide comparison with *S*. *spiritivorum* ATCC 33300

*S*. *deserti* is presumably well adapted to living in the high desert where it is exposed to high levels of solar UV irradiation, extreme cycles of high and low temperatures, and periodic desiccation throughout the year [41]. These extreme conditions cause both DNA- and oxidative protein damage in bacteria [42]. Bacterial adaptations to desert environments may include the protection of DNA and proteins from these damages, supplemented with efficient repair mechanisms to correct any damage still sustained [43–45]. To discover these important mechanisms, we compared the deduced proteome of strain ZW^T^ with that of *S*. *spiritivorum* ATCC 33300, a strain that was isolated in 1983 from vaginal secretions [1]. Protein orthology comparisons showed that 2,546 (75.1%) of the 3,391 predicted proteins of ZW^T^ have orthologs in *S*. *spiritivorum*. As expected, these conserved enzymes and pathways include those that are involved in sphingolipid metabolism: the biosynthesis of these lipids is a common distinguishing feature of the members of the genus *Sphingobacterium*. However, the large number of species-unique proteins of these two closely related bacteria may reflect their adaptations to their drastically different ecological niches (Table 3).

**Table 3:** Genome features of *S*. *deserti* ZW^T^ and comparison with *S*. *spiritivorum* ATCC 33300.

**Feature**	**ZW** ^T^	**ATCC 33300**
**Size (bp)**	4,615,818	5,232,948
**GC (mol%)**	42.6	39.9
**CDS (total)**	3,391	4,925
**tRNA**	48	44
**Genes with assigned function**	2,501	2,824
**Conserved hypothetical genes**	11	9
**Genes of unknown function**	84	-
**Hypothetical genes**	795	2,092

CDS, coding sequences.

Transport systems are key components for the tolerance of bacteria to their extreme environments [46]. For example, most of the known transport systems of extremophiles are sugar uptake systems which belong to the ABC family transporters [46]. Thus, ZW^T^ has 90 genes coding for transporters. Importantly, the *citH*, *zupT*, *yclN*, *metI*, *phnB*, *betT* and *dctA* genes encode putative transporters that are present in ZW^T^ but absent in *S*. *spiritivorum*. We hypothesize that these putative transporters may contribute to the adaptation of strain ZW^T^ to its desiccation-prone environment. Alternatively, these transporters may help strain ZW^T^ to import a wider range of nutrients from the desert soil that is poor in easily utilizable soil organic matter.

Trehalose is a nonreducing sugar with an α-1,1 linkage between two glucose moieties. Similar to other compatible osmolites, trehalose efficiently stabilizes proteins and lipid membranes, and thus protects against chemical and physical stresses in various organisms including bacteria, algae, yeast, fungi, plants and some invertebrates [47]. Strain ZW^T^ features several genes whose protein products are predicted to play a role in trehalose biosynthesis and thus may contribute to the desiccation tolerance of this organism [48]. Amongst these, two genes (h26-orf04608 for maltooligosyl trehalose trehalohydrolase and h26-orf04611 for maltooligosyl trehalose synthase) are present in ZW^T^ but are absent in *S*. *spiritivorum*.

Resistance to various toxic substances of abiotic or biotic origins also plays an important role in adaptations of microorganisms to extreme environments. Thus, the h26-orf01829 gene of strain ZW^T^ encodes an ortholog of the toxic anion resistance family protein TelA [49, 50]. TelA may facilitate the reduction, and the subsequent immobilization and detoxification of the metalloid oxyanion tellurite and other rare earth metal oxides and oxyanions [50]. The toxicity of the mobile and bioavailable tellurium oxyanions such as tellurite stem from their strong oxidizing ability, which interferes with many cellular enzymatic processes. The mechanism of the *telA*-encoded tellurite resistance remains unknown, although recent evidence suggests that the gene product is not associated with decreased uptake or increased efflux of tellurite [51]. No ortholog of TelA is encoded in the genome of *S*. *spiritovorum*.

An additional species-specific gene of strain ZW^T^, h26-orf02109, encodes a putative protein similar to the nisin resistance protein NSR [52]. Nisin is a 34-residue cationic antimicrobial peptide produced by *Lactococcus lactis* that is active against a wide range of Gram-positive bacteria. NSR inactivates nisin by proteolytic cleavage [53]. While sphingobacteria are not sensitive to nisin, other bacteriocins that are active against these bacteria may be produced by competitors of strain ZW^T^ in its native environment.

## Conclusions

Multiphase physiological, biochemical, and chemotaxonomic characterization, rDNA phylogeny, and genome sequencing was used to identify bacterial strain ZW^T^, isolated from the extreme arid environments of the Western Desert of China, as a novel species within the genus *Sphingobacterium*. Therefore, we propose ZW^T^ (= KCTC 32092^T^ = ACCC 05744^T^) as the type strain of *Sphingobacterium deserti* sp. nov. Whole genome sequencing and optical mapping of strain ZW^T^ was used to derive the first high quality, finished genome sequence assembly for any strain within the genus *Sphingobacterium*, Gram-negative bacteria that contain characteristic sphingolipids. Genome-wide comparisons with other sequenced sphingobacteria provided an extensive list of species-unique genes, some of which are proposed to contribute to the adaptation of *S*. *deserti* ZW^T^ to its extremely arid, inhospitable native environment.

## Supporting Information

S1 Fig Polar lipid profiles (including sphingolipids) of (a), strain ZW^T^, and (b), *S*. *spiritivorum*, detected by spraying the plates with a molybdatophosphoric acid reagent. PE, phosphatidylethanolamine; APL, unidentified aminophospholipid; SL, sphingolipid.
(DOCX)

S2 Fig Ninhydrin-positive spots of hexane-ether extracts from the hydrolysate of acetone-dried cells. 1, strain ZW^T^; 2, *S*. *spiritivorum* JCM 1277^T^; 3, authentic dihydrosphingosin. Silica gel 60 (Merck) plate. Mobile phase: Chloroform-methanol-water (65:25:4, v/v).
(DOCX)

S3 Fig Maximum parsimony phylogenetic tree based on 16S rRNA gene sequences, indicating the position of strain ZW^T^ in the *Sphingobacterium* genus. (DOCX)

S4 Fig Maximum likelihood phylogenetic tree based on 16S rRNA gene sequences, indicating the position of strain ZW^T^ in *Sphingobacterium* genus. (DOCX)

S5 Fig Neighbor-joining phylogenetic tree deduced from the orthologous proteins that occur in all 4 strains of the *Sphingobacterium* genus. (DOCX)

S1 Table Number of genes associated with the indicated COG functional categories. (DOCX)

S2 Table ANI values comparing genome identities of type strain ZW^T^ with the other four *Sphingobacterium* spp. for which genome sequences are available. (DOCX)
