# Supplementary material for: Whole-Genome Optical Mapping and Finished Genome Sequence of Sphingobacterium deserti sp. nov., a New Species Isolated from the Western Desert of China
Source: PLoS One. 2015 Apr 1;10(4):e0122254. doi: 10.1371/journal.pone.0122254 (PMC4382152; doi:10.1371/journal.pone.0122254)
Supplement: S1 Fig — PE, phosphatidylethanolamine; APL, unidentified aminophospholipid; SL, sphingolipid. (DOCX) [file pone.0122254.s001.docx]

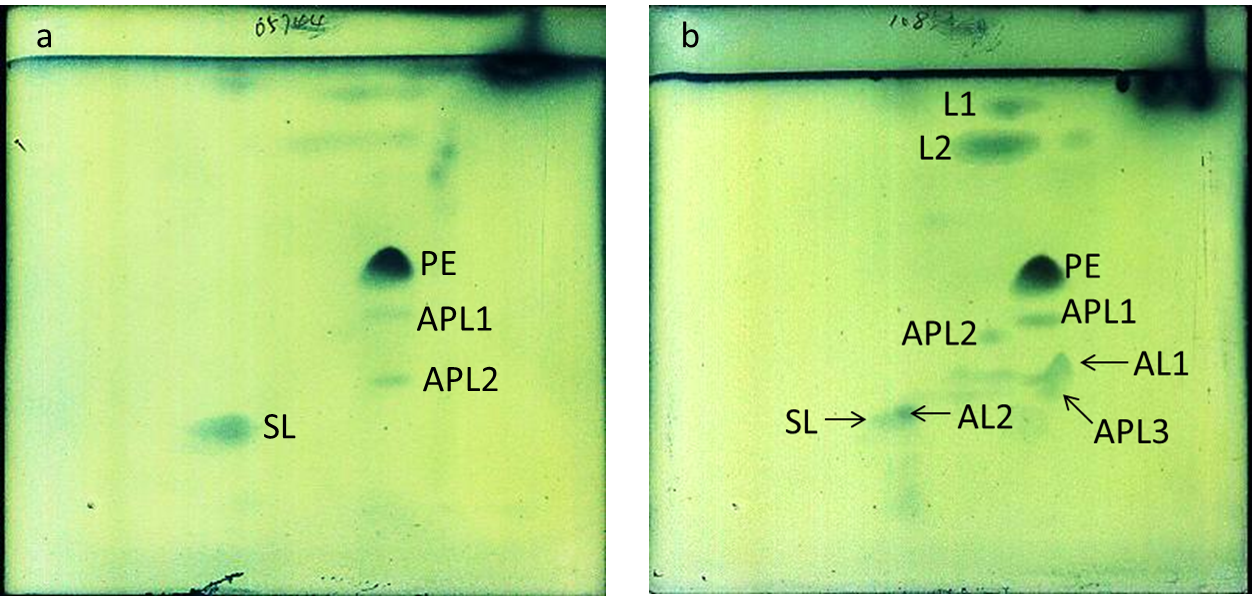


**Figure S1. Polar lipid profiles (including sphingolipids) of (a), strain ZW^T^, and (b), *S. spiritivorum*, detected by spraying the plates with a molybdatophosphoric acid reagent.** PE, phosphatidylethanolamine; APL, unidentified aminophospholipid；SL, sphingolipid
