# Supplementary material for: Whole-Genome Optical Mapping and Finished Genome Sequence of Sphingobacterium deserti sp. nov., a New Species Isolated from the Western Desert of China
Source: PLoS One. 2015 Apr 1;10(4):e0122254. doi: 10.1371/journal.pone.0122254 (PMC4382152; doi:10.1371/journal.pone.0122254)
Supplement: S2 Fig — 1, strain ZWT; 2, S. spiritivorum JCM 1277T; 3, authentic dihydrosphingosin. Silica gel 60 (Merck) plate. Mobile phase: Chloroform-methanol-water (65:25:4, v/v). (DOCX) [file pone.0122254.s002.docx]

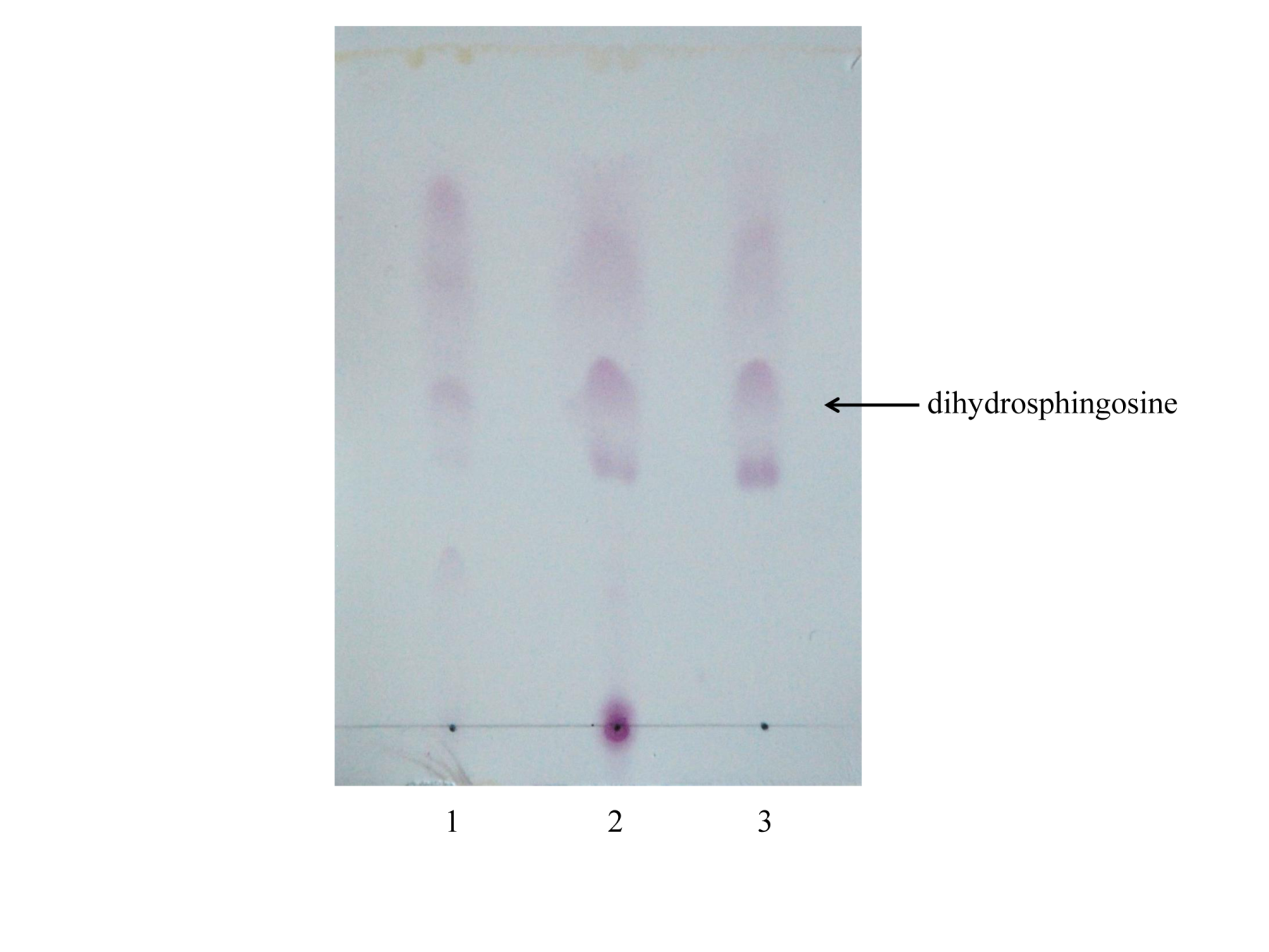
**Figure S2**. **Ninhydrin-positive spots of hexane-ether extracts from the hydrolysate of acetone-dried cells.** 1, strain ZW^T^; 2, *S. spiritivorum* JCM 1277^T^; 3, authentic dihydrosphingosine. Silica gel 60 (Merck) plate. Chloroform-methanol-water (65:25:4, v/v).
