# Supplementary material for: Whole-Genome Optical Mapping and Finished Genome Sequence of Sphingobacterium deserti sp. nov., a New Species Isolated from the Western Desert of China
Source: PLoS One. 2015 Apr 1;10(4):e0122254. doi: 10.1371/journal.pone.0122254 (PMC4382152; doi:10.1371/journal.pone.0122254)
Supplement: S5 Fig — (DOCX) [file pone.0122254.s005.docx]

**Figure S5**. **Neighbor-joining phylogenetic tree deduced from the orthologous proteins that occur in all 4 strains of the *Sphingobacterium* genus.**
