# Supplementary material for: Whole-Genome Optical Mapping and Finished Genome Sequence of Sphingobacterium deserti sp. nov., a New Species Isolated from the Western Desert of China
Source: PLoS One. 2015 Apr 1;10(4):e0122254. doi: 10.1371/journal.pone.0122254 (PMC4382152; doi:10.1371/journal.pone.0122254)
Supplement: S1 Table — (DOCX) [file pone.0122254.s006.docx]

**Table S1**. The ANI values of type strain ZW^T^ against the other 4 strains of the *Sphingobacterium* genus.

| Anlb(blast) | *S.deserti* ZWT | *S.spiritivorum* ATCC 33300 | *S.paucimobili*s HER1398 | *S.antarcticus* 4BY | *S.thalpophilum* DSM 11723 |
| --- | --- | --- | --- | --- | --- |
| *S.deserti* ZWT | 100.00 | 84.57 | 85.16 | 85.44 | 84.50 |
| *S.spiritivorum* ATCC 33300 | 84.69 | 100.00 | 85.01 | 86.27 | 85.55 |
| *S.paucimobilis* HER1398 | 84.94 | 84.86 | 100.00 | 86.42 | 84.98 |
| *S.antarcticus* 4BY | 85.41 | 86.19 | 86.43 | 100.00 | 92.16 |
| *S.thalpophilum* DSM 11723 | 84.40 | 85.48 | 85.05 | 92.18 | 100.00 |
