# Supplementary material for: Whole-Genome Optical Mapping and Finished Genome Sequence of Sphingobacterium deserti sp. nov., a New Species Isolated from the Western Desert of China
Source: PLoS One. 2015 Apr 1;10(4):e0122254. doi: 10.1371/journal.pone.0122254 (PMC4382152; doi:10.1371/journal.pone.0122254)
Supplement: S2 Table — (DOCX) [file pone.0122254.s007.docx]

| **Table S2**. Number of genes associated with the indicated COG functional categories. | | | |
| --- | --- | --- | --- |
| **Code** | **value** | **% age** | **Description** |
| J | 128 | 4 | Translation, ribosomal structure and biogenesis |
| A | 0 | 0 | RNA processing and modification |
| K | 142 | 4.5 | Transcription |
| L | 114 | 3.6 | Replication, recombination and repair |
| B | 0 | 0 | Chromatin structure and dynamics |
| D | 17 | 0.5 | Cell cycle control, cell division, chromosome partitioning |
| Y | 0 | 0 | Nuclear structure |
| V | 45 | 1.4 | Defense mechanisms |
| T | 121 | 3.8 | Signal transduction mechanisms |
| M | 191 | 6 | Cell wall/membrane/envelope biogenesis |
| N | 10 | 0.3 | Cell motility |
| Z | 0 | 0 | Cytoskeleton |
| W | 0 | 0 | Extracellular structures |
| U | 17 | 0.5 | Intracellular trafficking, secretion, and vesicular transport |
| O | 95 | 2.9 | Posttranslational modification, protein turnover, chaperones |
| C | 122 | 3.8 | Energy production and conversion |
| G | 143 | 4.5 | Carbohydrate transport and metabolism |
| E | 175 | 5.5 | Amino acid transport and metabolism |
| F | 55 | 1.7 | Nucleotide transport and metabolism |
| H | 94 | 2.9 | Coenzyme transport and metabolism |
| I | 88 | 2.8 | Lipid transport and metabolism |
| P | 141 | 4.4 | Inorganic ion transport and metabolism |
| Q | 24 | 0.8 | Secondary metabolites biosynthesis, transport and catabolism |
| R | 249 | 7.8 | General function prediction only |
| S | 165 | 5.2 | Function unknown |
| - | 1046 | 32.8 | Not in COGs |
